# Biomimetic calcium carbonate nanoparticles delivered IL-12 mRNA for targeted glioblastoma sono-immunotherapy by ultrasound-induced necroptosis

**DOI:** 10.1186/s12951-022-01731-z

**Published:** 2022-12-10

**Authors:** Pengxuan Zhao, Yu Tian, Yongping Lu, Jun Zhang, Anyu Tao, Guangya Xiang, Yani Liu

**Affiliations:** 1 Department of Medical Ultrasound, Tongji Hospital, Tongji Medical College, Huazhong University of Science and Technology, Wuhan, 430030 China; 2 School of Pharmacy, Tongji Medical College, Huazhong University of Science and Technology, Wuhan, 430030 China; 3 School of Pharmacy, Hainan Medical University, Haikou, 571199 China; 4 Jiangsu Hengrui Pharmaceuticals Co. Ltd, Lianyungang, China; 5 Department of Ultrasound, The Affiliated Hospital of Yunnan University, Kunming, 650021 China

**Keywords:** Biomimetic nanoparticles, mRNA delivery, Glioblastoma targeting, Necroptosis, Sono-immunotherapy

## Abstract

Glioblastoma (GBM) is the most aggressive brain tumor, which owns the characteristics of high recurrence, low survival rate and poor prognosis because of the existence of blood brain barrier (BBB) and complicated brain tumor microenvironment. Currently, immunotherapy has attracted much attention on account of favorable therapeutic effect. In this study, we designed a cRGD-modified cancer cell membrane (CM) coated calcium carbonate nanoparticle to deliver interleukin-12 messenger RNA (IL-12 mRNA@cRGD-CM-CaCO_3_ NPs). The cRGD-modified CM as the shell can endow the nanoparticles with BBB crossing and tumor homing/homotypic targeting effect in the brain tumor microenvironment. IL-12 mRNA-loaded calcium carbonate nanoparticles as the core allow synergistic immunotherapy of necroptosis-induced immune response and IL-12 mRNA transfection under ultrasound irradiation. The as-prepared biomimetic nanoparticles showed superior target and immunotherapeutic outcomes, suggesting that this biomimetic nanoplatform provides a feasible strategy for promoting BBB-penetrating and antitumor immunity.

**Supplementary Information:**

The online version contains supplementary material available at 10.1186/s12951-022-01731-z.

## Introduction

Glioblastoma multiforme (GBM) is the most common malignant primary brain tumor, with a median survival less than 2 years [1]. The standard GBM therapy include maximal surgical resection later on with chemical therapy and radiation therapy. Unfortunately, GBM always grow into normal brain tissue, so it is almost impossible to remove the entire tumor [2]. More important, the presence of blood–brain barrier (BBB) prevent majority of drugs into the brain [3]. Based on these, most of the GBM patients will eventually relapse though suffering repeated surgical resection and chemo/radiotherapy [4]. Therefore, novel and efficient strategies are urgently needed to improve the treatment outcome of GBM.

Nowadays, immunotherapy has achieved promising clinical outcomes in a diversity of solid tumors, such as advanced melanoma and non-small cell lung cancer [5]. It is believed that successful immunotherapy requires a self-sustaining “cancer-immunity cycle”, that is, immunogenic cell death (ICD) and activated dendritic cells (DCs) initially induce T-cell responses in draining lymph nodes. Then these T cells and other immune cells migrate to the tumor site, where promoting continued tumor cell killing and remodeling the tumor microenvironment [6, 7]. Hence, a variety of interlinked events are necessary for initiating this cycle, such as ICD induction, DCs activation, immune cells recruitment, proinflammatory factors generation and so on. Among them, the key is to induce ICD. For ICD induction, the most common method is utilizing certain chemical drugs to cause immunogenic apoptosis, which release tumor-associated antigens (TAAs) and damage-associated molecular patterns (DAMPs) to activate tumor-specific immune response [8, 9]. However, the immunogenicity of released TAAs and DAMPs would significantly decrease during the apoptosis process, due to this process is often accompanied by intracellular oxidation and proteolysis, ultimately leading to limited antitumor immunity [10, 11]. As a consequence, it is urgent to develop efficient ICD inducers that could avoid the degradation of released TAAs and DAMPs.

Necroptosis, characterized by plasma membrane disintegration, is induced through specific stimulus such as mechanical stress and temperature variation [12]. In contrast to apoptosis, necroptosis process does not cause intracellular oxidation and proteolysis, so the released TAAs and DAMPs could keep their biological activity [13]. As a result, necroptosis could active immune response more effectively than immunogenic apoptosis. Previous studies have reported that calcium carbonate nanoparticles (CaCO_3_ NPs) could generate CO_2_ bubbles in tumoral lysosome acidic condition, and induce cavitation-mediated necroptosis under ultrasound (US) irradiation [14, 15]. Besides, CaCO_3_ NPs themselves are suitable vehicles for the delivery of small molecule drugs, genes and proteins [16, 17]. Considering messenger RNA (mRNA) represents a new type of therapeutics, and interleukin-12 (IL-12) is a promising candidate for cancer immunotherapy through activating T cell functions but lack of intravenous delivery approaches [18, 19]. Hence, we hypothesized that using CaCO_3_ NPs encapsulating mRNA encoded IL-12 and combined with US irradiation, could be a promising synergetic strategy to enhance antitumor immunity.

How to deliver CaCO_3_ NPs across the BBB and into the tumor cells is another challenge for effective GBM immunotherapy. Cyclic Arg-Gly-Asp (cRGD), as a well characterized peptide that could bind to αvβ3 integrin overexpressed in GBM neovasculature, has been widely used for the BBB penetrating [20]. Nevertheless, complicated brain tumor microenvironment (containing not only brain tumor cells, but also fibroblasts, astrocytes and microglias) still hinder the brain tumor cells accumulation of cRGD-modified nanoparticles [21, 22]. Recently, cell membrane (CM) coating nanotechnology has garnered much attention for constructing biomimetic nanoparticles, which could assist GBM cell target through tumor homing and homotypic targeting capacities because of the complete replication of surface antigens from the GBM CM [21, 23, 24]. Thus, it is envisioned that combined of cRGD modification and CM coating in CaCO_3_ NPs might further improve the GBM cell targeted ability after crossing the BBB, thereby ensuring the necroptosis induction by in situ CO_2_ bubbles production under US and IL-12 mRNA translation in brain tumor cells.

**Scheme 1:**
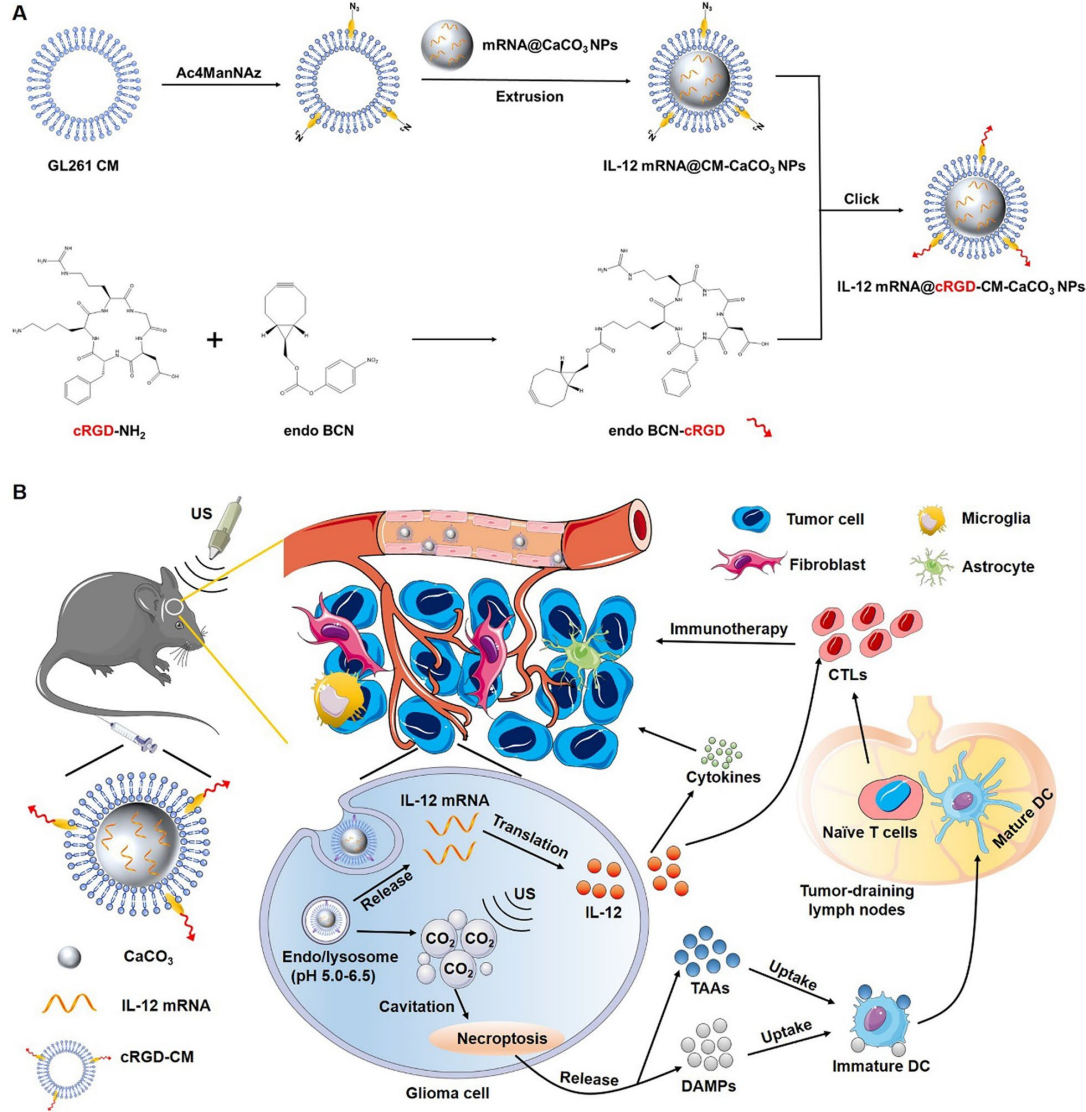
**A** Illustration for the preparation process of IL-12 mRNA@cRGD-CM-CaCO_3_ NPs. **B** Proposed mechanism of IL-12 mRNA@cRGD-CM-CaCO_3_ NPs for BBB penetration, tumor microenvironment navigation and sono-immunotherapy synergistic anti-tumor effects in GBM

Herein, we designed a CM coated CaCO_3_ NPs, which CaCO_3_ NPs loaded with IL-12 mRNA as the core and cRGD-labeled GBM CM as the shell (named as IL-12 mRNA@cRGD-CM-CaCO_3_ NPs, Scheme 1A). After intravenous injection, IL-12 mRNA@cRGD-CM-CaCO_3_ NPs directly entered GBM cell through BBB crossing and tumor homing/homotypic targeting capabilities (Scheme 1B). Firstly, CaCO_3_ NPs decomposed and generated CO_2_ gas in lysosome environment. Subsequently, CO_2_ bubbles collapsed and induced necroptosis of GBM cells by cavitation effect under US irradiation. Then, necroptosis released TAAs and DAMPs can be taken up and processed by DCs. Once activation, mature DCs would present antigens to T cells and trigger subsequent antitumor immunity. Meanwhile, loaded IL-12 mRNA was translated to IL-12 in the cytoplasm, which could stimulate the proliferation and activation of cytotoxic T lymphocytes (CTLs), as well as the production of cytokines. Based on the in vitro and in vivo results, we revealed that IL-12 mRNA@cRGD-CM-CaCO_3_ NPs were able to traverse through the BBB and target GBM cells. Furthermore, strong synergistic immunotherapy was achieved through the combination of acoustic cavitation-mediated necroptosis and IL-12 mRNA transfection.

## Results and discussion

### The preparation and characterization of nanoparticles

**Fig. 1:**
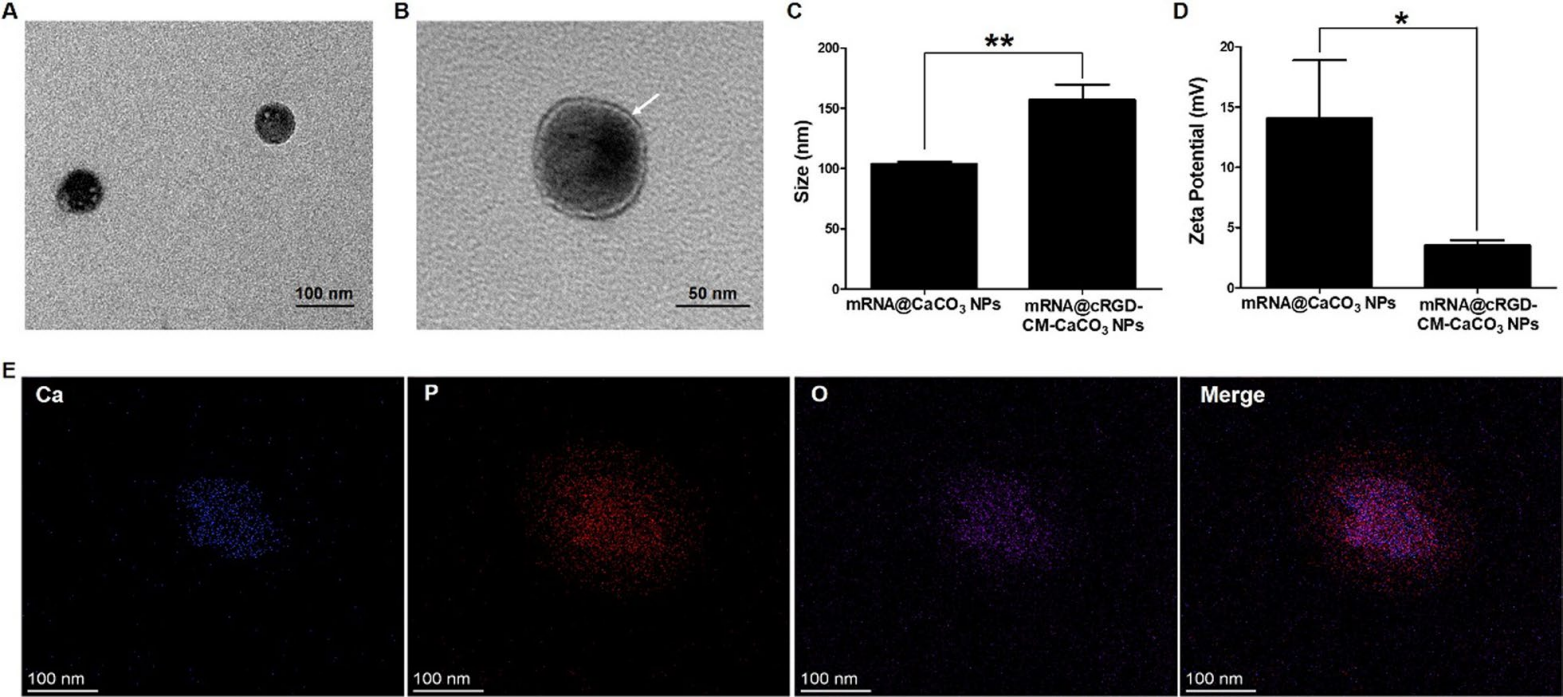
Preparation and characterization of mRNA@cRGD-CM-CaCO_3_ NPs. **A** TEM image of mRNA@CaCO_3_ NPs cores. **B** TEM image of mRNA@CaCO_3_ NPs cores coated with cRGD-CM shells (mRNA@cRGD-CM-CaCO_3_ NPs). Arrows show the shells surrounding the cores. **C**, **D** Size and zeta potential measurement of mRNA@CaCO_3_ NPs and mRNA@cRGD-CM-CaCO_3_ NPs. (E) Energy-dispersive X-ray spectroscopy (EDS) elemental mapping of mRNA@cRGD-CM-CaCO_3_ NPs. Data are expressed as mean ± SEM (n = 5). *P < 0.05, **P < 0.01

Firstly, CaCO_3_ NPs loaded with mRNA (mRNA@CaCO_3_ NPs) were prepared through a reverse microemulsion method. Transmission electron microscopy (TEM) observed that CaCO_3_ NPs were spherical in shape with a size of about 60 nm (Fig. 1A). Then, cell membrane (CM) was derived from GL261 cells through repeated freeze–thaw process. To prepare cRGD-labeled CM (cRGD-CM), GL261 cells were pre-treated with N-azidoacetylmannosamine-tetraacylated (Ac4ManNAz) to attach azide group on the cell surface[25]. After that, for the preparation of CM coated CaCO_3_ NPs (mRNA@CM-CaCO_3_ NPs), CM and mRNA@CaCO_3_ NPs were mixed and co-extruded by a 200 nm polycarbonate membrane. Finally, click reaction was used to modify cRGD on the surface of mRNA@CM-CaCO_3_ NPs, which was produced between the azide groups of cell surface and the alkyne groups of the pre-synthesized endo-bicyclo[6.1.0]nonyne(BCN)-cRGD (endo-BCN-cRGD). The successful production of endo-BCN-cRGD was verified through mass spectroscopy (Additional file 1: Fig. S1). After cRGD attached on the surface of mRNA@CM-CaCO_3_ NPs, mRNA@cRGD-CM-CaCO_3_ NPs were finally prepared. As shown in Fig. 1B, obvious core–shell structure (a visible shell layer of ≈ 8 nm) was observed in mRNA@cRGD-CM-CaCO_3_ NPs, indicating successful CM fusion. The CM coating was further verified through the size and zeta potential changes detected through dynamic light scattering (DLS). An increase of average hydrodynamic diameters from 104 nm (mRNA@CaCO_3_ NPs) to 157 nm (mRNA@cRGD-CM-CaCO_3_ NPs) was observed (Fig. 1C). The larger sizes measured through DLS than TEM could attribute to the surface hydration of NPs in DLS detections. The zeta potential was decreased from 14.1 mV to 3.5 mV after CM coated (Fig. 1D). Meanwhile, the CM coating and mRNA encapsulation can be also proved by elemental mapping (Fig. 1E), where the P element, as a representative element of CM and mRNA, was well distributed both inside and outside the Ca element. In addition, the encapsulation efficiency of mRNA in mRNA@cRGD-CM-CaCO_3_ NPs was approximately 70% at the loading capacity of nearly 2% (mRNA weight/mRNA@cRGD-CM-CaCO_3_ NPs weight). These results proved that CM was successfully coated in the surface of mRNA@CaCO_3_ NPs.

**Fig. 2:**
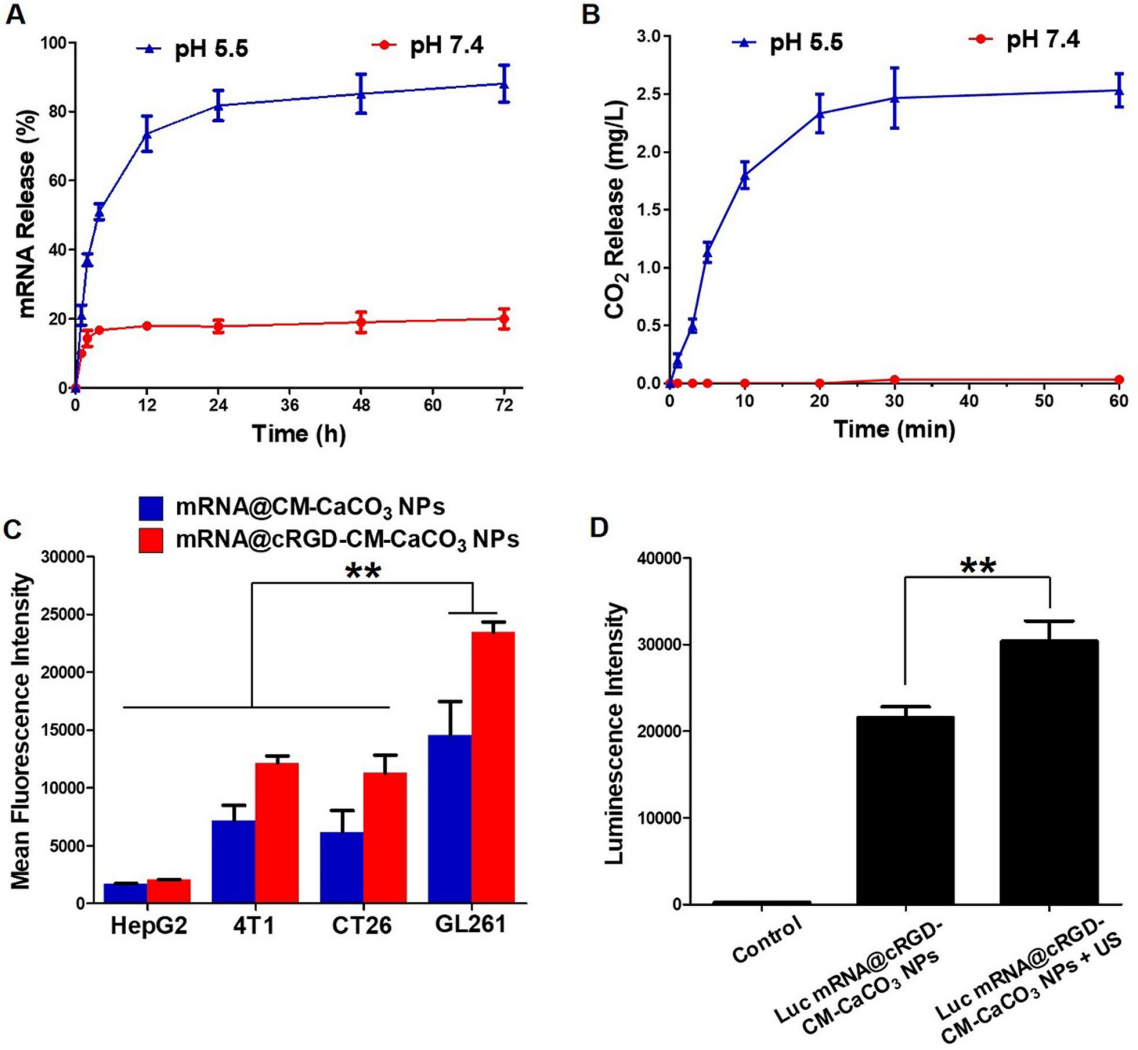
**A** Cumulative mRNA release from mRNA@cRGD-CM-CaCO_3_ NPs. **B** The quantification of CO_2_ generation from mRNA@cRGD-CM-CaCO_3_ NPs. **C** Homotypic targeting through fluorescence measurement of nanoparticles incubated in different cell lines. **D** Luminescence intensity of nanoparticles. Data are expressed as mean ± SEM (n = 5). **P < 0.01

To confirm that mRNA@cRGD-CM-CaCO_3_ NPs could arrive in the tumor site before decomposition, the pH-dependent release experiment of mRNA from nanoparticles was performed. As shown in Fig. 2A, less amount of Cy3-labelled mRNA (Cy3-mRNA) was released from mRNA@cRGD-CM-CaCO_3_ NPs at neutral conditions, demonstrating that CaCO_3_ NPs were stable in the systemic circulation. By contrast, at pH 5.5 condition, faster release of mRNA was observed, and nearly 90% of mRNA was released after 72 h. These results demonstrated the pH-activated decomposition of CaCO_3_ NPs. The quantitative analysis of CO_2_ gas generation was further evaluated (Fig. 2B), almost no CO_2_ gas was produced from mRNA@cRGD-CM-CaCO_3_ NPs at neutral conditions. In contrast, a considerable amount of CO_2_ was generated at pH 5.5 condition.

### Cellular uptake and transfection of mRNA@cRGD-CM-CaCO_3_ NPs

To test whether cRGD-CM-CaCO_3_ NPs could effectively deliver mRNA into brain tumor GL261 cells. Cellular uptake and luciferase transfection assay were performed. We first used Cy3-mRNA to detect the cellular uptake efficiency in different cell lines including HepG2, 4T1, CT26 and GL261 cells through flow cytometry (Fig. 2C). When comparison was made among all 4 cell lines, the mean fluorescence intensity of GL261 cells treated with mRNA@CM-CaCO_3_ NPs or mRNA@cRGD-CM-CaCO_3_ NPs were significantly stronger, suggesting that GL261 CM coating could assist CaCO_3_ NPs enter into GL261 cells through the homotypic targeting effect. Furthermore, when comparison was made in each cell line, the mean fluorescence intensity was stronger in cRGD-labeled group (mRNA@cRGD-CM-CaCO_3_ NPs) compared with CM coated only group (mRNA@CM-CaCO_3_ NPs), which proved that cRGD played a significant role in facilitating the cellular uptake of nanoparticles. Subsequently, we used mRNA encoding luciferase (Luc mRNA) to evaluate the transfection efficiency of mRNA@cRGD-CM-CaCO_3_ NPs. According to Fig. 2D, Luc mRNA@cRGD-CM-CaCO_3_ NPs were able to transfect GL261 cells with a high luminescence intensity. Additionally, the transfection efficiency of Luc mRNA was further improved after US irradiation (2776 Intellect Mobile Ultrasound Device, Chattanooga, USA), which might due to the enhanced gene delivery efficiency through US-mediated acoustic cavitation and sonoporation effect [26].

### In Vitro immunogenic necroptosis effect of nanoparticles

**Fig. 3:**
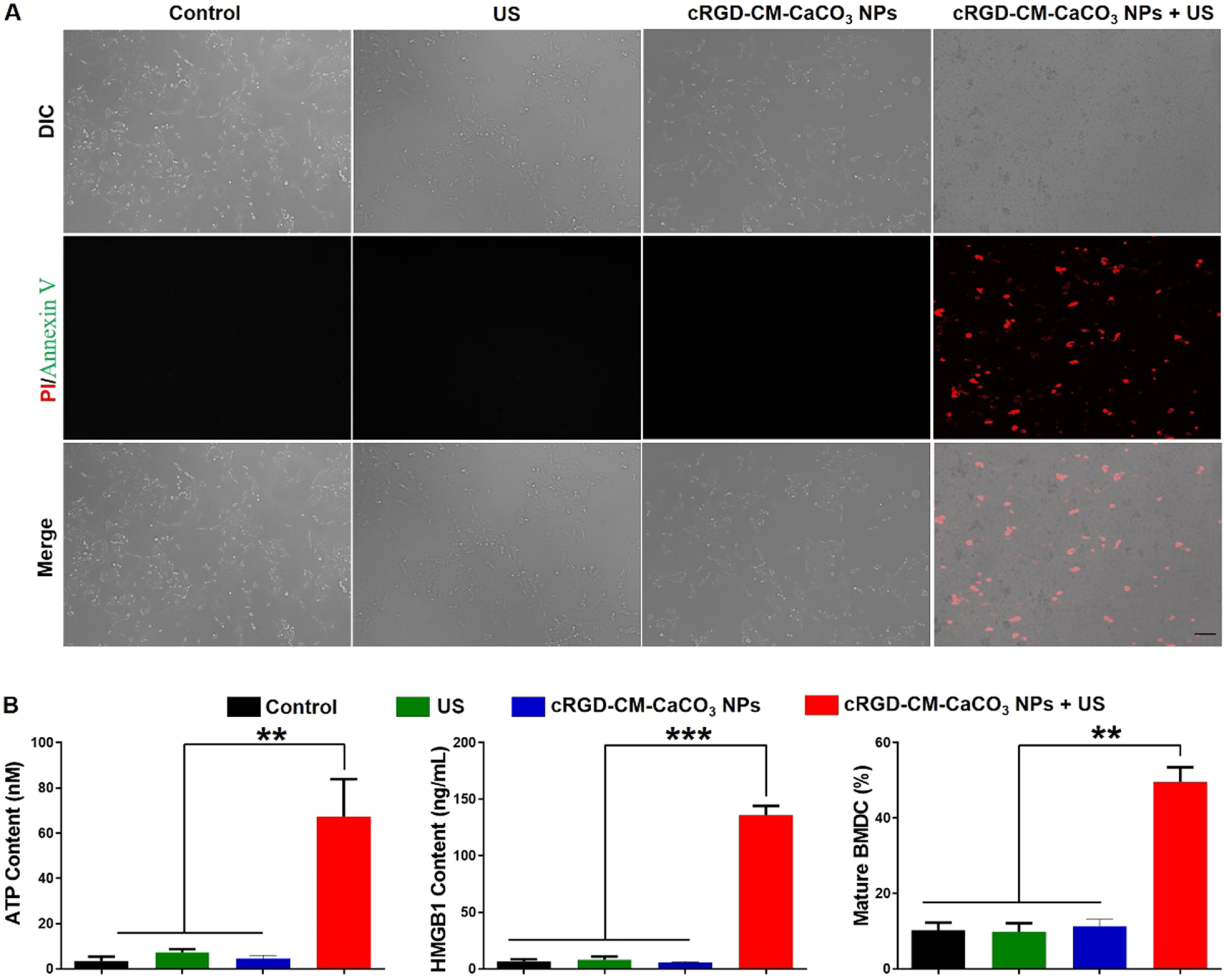
cRGD-CM-CaCO_3_ NPs-induced necroptosis and DAMPs release. **A** Fluorescence microscope images of annexin V (green fluorescence)/PI (red fluorescence)-stained GL261 cells. Scale bar, 100 µm. **B** Release of ATP, HMGB1 and bone marrow-derived dendritic cells (BMDCs) maturation after different treatments. Data are expressed as mean ± SEM (n = 5). **P < 0.01, ***P < 0.001

Necroptosis is characterized by membrane rupture and cytoplasmic swelling [27]. To verify the cell death mechanism, the annexin V/propidium iodide (PI) assay was performed in GL261 cells [10, 28]. As illustrated by Fig. 3A, when the cells were only treated with US irradiation or cRGD-CM-CaCO_3_ NPs, no significant changes in the morphology and no fluorescent signals from annexin V/PI were observed, which proved that US irradiation or cRGD-CM-CaCO_3_ NPs alone did not cause obvious damage to GL261 cells. Whereas, cRGD-CM-CaCO_3_ NPs plus US-treated cells displayed a loss in their cell morphology, demonstrating that the cell membrane was damaged due to the US-mediated cavitation effect. Moreover, the membrane rupture induced the leakage of membrane fragments, cytosolic components and chromatins [29]. These results validated that the combination of cRGD-CM-CaCO_3_ NPs and US irradiation could induce necroptosis of GL261 cells. Encouraged by the above data, released DAMPs (including HMGB1 and ATP) were also evaluated. cRGD-CM-CaCO_3_ NPs + US group significantly improved the extracellular secretion of HMGB1 and ATP compared with other groups. As a result, the DCs maturation frequency of cRGD-CM-CaCO_3_ NPs + US group was highest and up to 49.6% (Fig. 3B).

Next, cRGD-CM-CaCO_3_ NPs were loaded with IL-12 mNRA and the in vitro cytotoxicity was performed by methyl thiazolyl tetrazolium (MTT) assay. As expected (Additional file 1: Fig. S2), no significant cytotoxic effect was observed in cRGD-CM-CaCO_3_ NPs group. In contrast, cRGD-CM-CaCO_3_ NPs + US group and IL-12 mRNA@cRGD-CM-CaCO_3_ NPs group exhibited moderate cytotoxic effect, which was attributed to acoustic cavitation or the efficacy of IL-12 mRNA respectively. Notably, IL-12 mRNA@cRGD-CM-CaCO_3_ NPs + US group showed the strongest cytotoxicity and killed nearly 70% of the cells, implying that the combination treatment of IL-12 mRNA@cRGD-CM-CaCO_3_ NPs and US irradiation could increase in vitro antitumor effect.

Overall, these results proved that US-mediated necroptosis leaded to the release of DAMPs, which induced the DCs maturation and enhanced antitumor immunity.

### *In vivo* imaging and safety evaluation

**Fig. 4:**
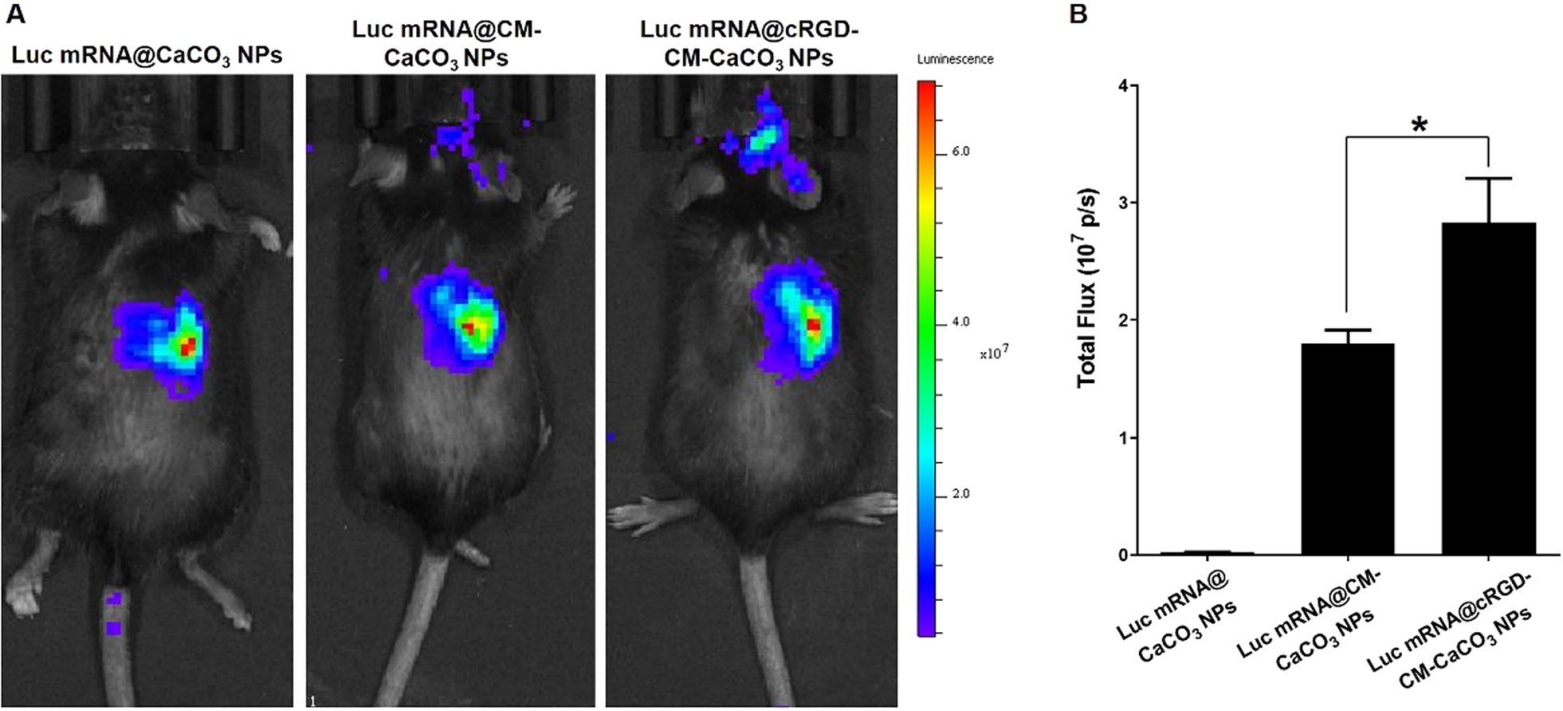
In vivo luminescence imaging results after different injections in mice brain tumor model (**A**) and quantitative bioluminescence signal intensity (**B**). Data are expressed as mean ± SEM (n = 3). *P < 0.05

To verify the brain tumor-targeted of cRGD-CM-CaCO_3_ NPs in vivo, an intracranial orthotopic glioblastoma (GL261) mice model was used. Luc mRNA@ CaCO_3_ NPs, Luc mRNA@CM-CaCO_3_ NPs or Luc mRNA@cRGD-CM-CaCO_3_ NPs were intravenously injected at an mRNA dose of 0.25 mg/kg. After 6 h, we measured the bioluminescence signals by a IVIS imaging system (Fig. 4A). Most of the Luc mRNA@ CaCO_3_ NPs accumulated in the liver, once coated with CM, part of the Luc mRNA@CM-CaCO_3_ NPs were found in the brain tumor site. More importantly, after cRGD decorated, Luc mRNA@cRGD-CM-CaCO_3_ NPs displayed nearly 1.6-fold higher bioluminescence signal intensity than CM coated alone group (Luc mRNA@CM-CaCO_3_ NPs) in the glioma area (Fig. 4B). These results demonstrated that CM coated contribute to brain tumor targeting, and the cRGD modification can further enhance the targeting capability.

Next, we evaluated the toxicity of the nanoparticles in healthy C57BL/6 mice. The measurement of blood biochemistry parameters and HE staining of major organs were performed after treated with PBS, IL-12 mRNA@CaCO_3_ NPs, IL-12 mRNA@CaCO_3_ NPs + US, IL-12 mRNA@cRGD-CM-CaCO_3_ NPs or IL-12 mRNA@cRGD-CM-CaCO_3_ NPs + US. Blood urea nitrogen (BUN) is commonly used for assessing renal function [30]. Aspartate aminotransferase (AST) and alanine aminotransferase (ALT) are effective predictors of liver pathology [31]. As shown in Additional file 1: Fig. S3, there was no obvious differences in BUN, AST and ALT levels among all the groups, proving that no significant renal and liver toxicity after nanoparticles treating. Moreover, no apparent histopathological changes were found in the major organs through HE staining (Additional file 1: Fig. S4). For further verifying the safety of US irradiation to the brain, HE staining of brain was performed after treated with PBS or US irradiation in healthy C57BL/6 mice. And no apparent histopathological changes in brain were found after US irradiation (Additional file 1: Fig. S5), which proving that therapeutic US irradiation was safe for the normal brain tissues. All these results demonstrated that CaCO_3_ NPs plus US irradiation can serve as a safe strategy for tumor therapy.

### *In vivo* anti-glioma activity

**Fig. 5:**
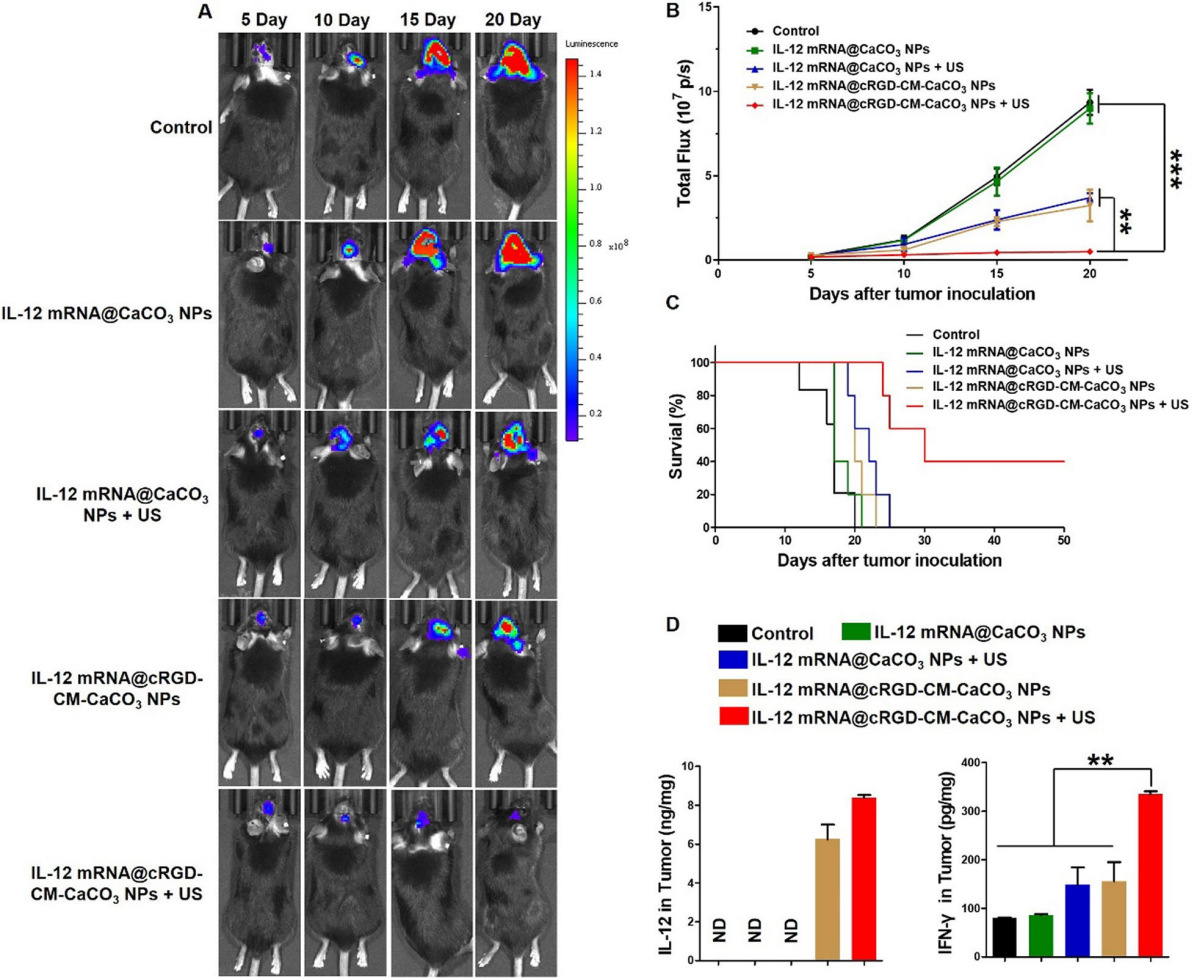
In vivo anti-glioma activity of IL-12 mRNA@cRGD-CM-CaCO_3_ NPs. **A** Representative bioluminescence images of GL261-Luc glioma-bearing mice in different groups. **B** Quantitative analysis of bioluminescence signal intensity. **C** Survival curve for the mice (n = 5 mice per group). **D** IL-12 and IFN-γ expression in tumors from immunized mice. Data are expressed as mean ± SEM (n = 5). ND, not detected. **P < 0.01, ***P < 0.001

Encouraged by the excellent antitumor effects in vitro and brain-targeting ability in vivo of cRGD-CM-CaCO_3_ NPs, we investigated antitumor efficacy of the nanoparticles in vivo by an orthotopic GL261-Luc glioma mouse model. As illustrated by Fig. 5A, B, IVIS Spectrum showed that rapid tumor growth in the PBS or IL-12 mRNA@CaCO_3_ NPs treated group. Whereas moderately restricted cancer growth was observed in IL-12 mRNA@CaCO_3_ NPs + US group and IL-12 mRNA@cRGD-CM-CaCO_3_ NPs group. Furthermore, the bioluminescence signals of IL-12 mRNA@cRGD-CM-CaCO_3_ NPs + US group was obviously weaker than any other group, indicating the strongest antitumor effect. In addition, survival study also proved that the combination of IL-12 mRNA@cRGD-CM-CaCO_3_ NPs and US irradiation can extend mice survival and lead to a 40% durable cure rate (Fig. 5C). The body weight of mice was greatly affected by different therapies, which is analogous to the trend of survival rate (Additional file 1: Fig. S6).

**Fig. 6:**
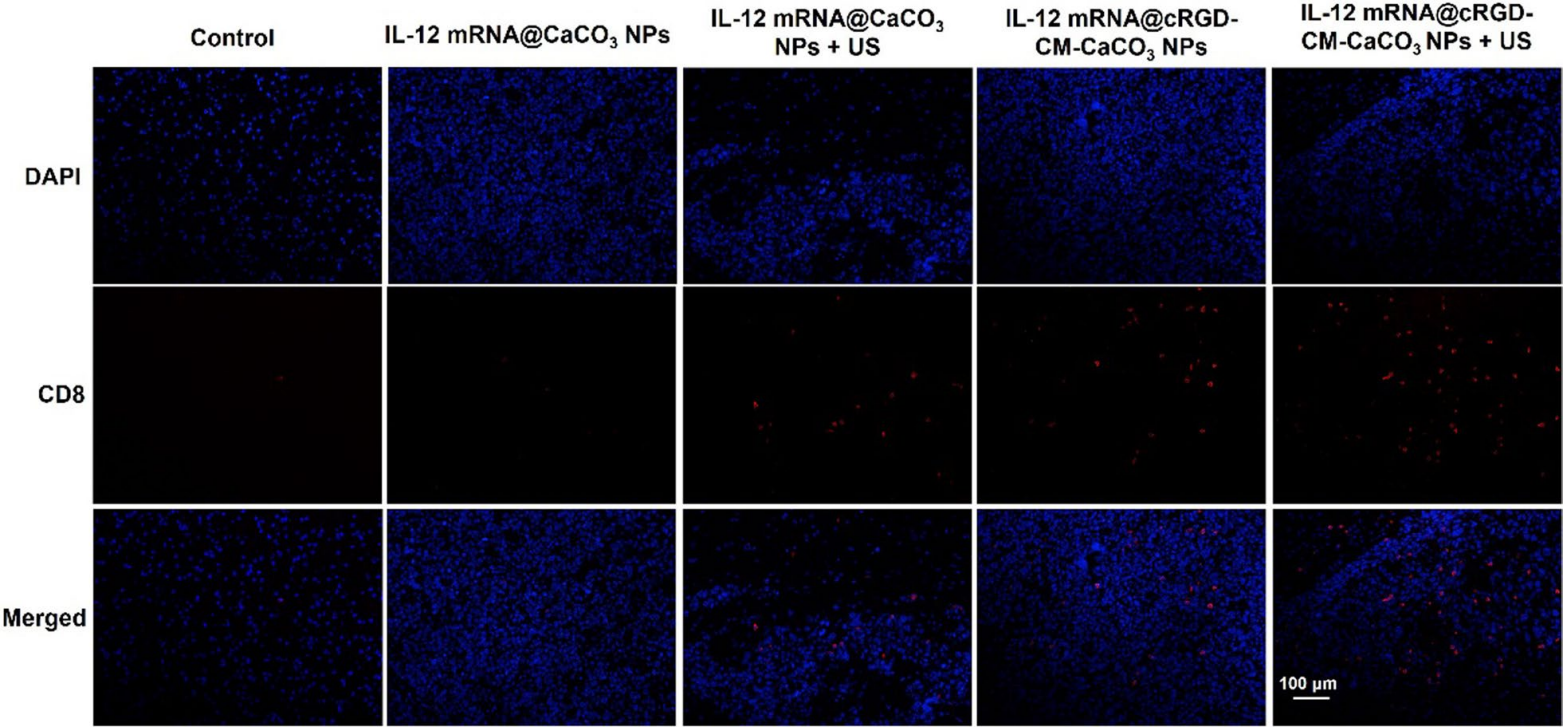
CD8 (red fluorescence) T cells in tumor tissues after different treatments. Scale bar, 100 µm

Importantly, IL-12 mRNA@cRGD-CM-CaCO_3_ NPs treatment plus US significantly increased the expression of IL-12 in brain tumor sections, as well as the IFN-γ production, which is induced by IL-12 directly (Fig. 5D) [32]. Moreover, IL-12 mRNA@cRGD-CM-CaCO3 NPs + US group had the largest proportion of CD8 + T cells in tumors compared with other groups (Fig. 6). Altogether, these results indicated that the anti-glioma immune response by IL-12 mRNA@cRGD-CM-CaCO_3_ NPs could be amplified through US-mediated necroptosis.

## Conclusion

In summary, we have developed a novel biomimetic nanoparticle, including a shell of cRGD-modified cell membrane and a core of CaCO_3_ NPs loaded with IL-12 mRNA (IL-12 mRNA@cRGD-CM-CaCO_3_ NPs). Such a design of shell conferred BBB crossing and tumor homing/homotypic targeting abilities to nanoparticles, thus further promoting the brain tumor targeting. When exposed to US, the CaCO_3_ NPs core could induce necroptosis by CO_2_ bubbles-mediated cavitation effect, resulting in DAMPs released and DCs maturation. Combined with IL-12 mRNA, a superior antitumor activity against GBM was induced both in vitro and in vivo, which was attributed to the enhanced tumor targeting and DCs maturation, as well as the CTLs stimulation. Taken together, our strategy provides a platform for ultrasound-immune synergistic therapy of brain tumors.

## Supplementary Information

**Additional file 1.** Supplementary materials.

## Data Availability

All data generated or analyzed during this study are included in this manuscript.
