# Supplementary material for: Biomimetic calcium carbonate nanoparticles delivered IL-12 mRNA for targeted glioblastoma sono-immunotherapy by ultrasound-induced necroptosis
Source: J Nanobiotechnology. 2022 Dec 10;20:525. doi: 10.1186/s12951-022-01731-z (PMC9741778; doi:10.1186/s12951-022-01731-z)
Supplement: Supplementary file 1 — Additional file 1. Supplementary materials. [file 12951_2022_1731_MOESM1_ESM.docx]

**Additional file**

**Experimental Section**

***Materials***

Calcium chloride, phosphotungstic acid, sodium carbonate, dimethyl sulfoxide (DMSO), cyclohexane, triton-X100, hexanol, triethylamine (TEA), chloroform, dimethylformamide (DMF), 3-(4,5-dimethylthiazol-2-yl)-2,5-diphenyltetrazolium bromide (MTT) and other chemicals were purchased from Sigma-Aldrich Chemical Co (St. Louis, MO, USA). Trelief^®^ solution was purchased from Tsingke Biological Technology. cRGD-NH_2_, endo-bicyclo[6.1.0]nonyne (endo-BCN), N-azidoacetylmannosamine-tetraacylated (Ac4ManNAz) were purchased from Xi'an ruixi Biological Technology Co. Ltd. Cell culture dishes/plates, round coverslips, and centrifuge tubes were obtained from NEST Biotechnology Co. Ltd. (Wuxi, China). Mouse high mobility group protein 1 (HMGB1) and adenosine triphosphate (ATP) enzyme-linked immunosorbent assay (ELISA) kits were purchased from Elabscience Biotechnology Co. Ltd. Mouse interferon-γ (IFN-γ) and IL-12 ELISA kits were purchased from Abbkine Scientific (Wuhan, China). Aspartate transaminase (ALT), aspartate aminotransferase (AST), blood urea nitrogen (BUN), creatinine (CREA) colorimetric assay kits were purchased from Solarbio kit (Beijing, China).

Fetal bovine serum (FBS), RiboGreen RNA reagent and Dulbecco’s modified Eagle’s high glucose medium (DMEM) were purchased from Thermo Fisher Scientific (Chicago, IL, USA). Mouse glioblastoma GL261 cells and GL261 cells transformed with luciferase gene (GL261-Luc) were kind gifts from Prof. Xiaobing Jiang (Union Hospital, Tongji Medical College of Huazhong University of Science and Technology). IL-12 mRNA and Cy3-labelled mRNA were synthesized by GenePharma (Shanghai, China). mRNA encoding firefly luciferase (FLuc mRNA) was purchased from APExBIO Technology LLC. luciferase assay substrate D-Luciferin, potassium salt was from Yeasen Biotechnology (Shanghai) Co. Ltd. US irradiation was carried out through using a 2776 Intellect Mobile Ultrasound Device (Chattanooga, USA). For *in vitro* experiments, US irradiation (1 MHz, 50% duty cycle, 0.5 W/cm^2^, 1 min) was performed at the 4^th^ hour after nanoparticles treat. For *in vivo* experiments, US irradiation (1 MHz, 50% duty cycle, 1.0 W/cm^2^, 3 min) was performed at the 12^th^ hour after nanoparticles treat.

***Preparation of azide-modified GL261 cell membrane (CM)***

GL261 CM were prepared according to previous literature^1^. GL261 cells were cultured on 10 cm glass bottom dish. To attach azide group onto the cell surface, Ac4ManNAz was added to the culture medium to achieve a concentration of 50 μM. The CM were obtained through repeated freeze-thaw process. First, GL261 cells were collected and washed with PBS for three times. Then they were dispersed in hypotonic lysing buffer and kept at 4 °C overnight. Subsequently, the solution was frozen at -80 °C, then thawed at 37 °C. Such cycle of freezing-thawing repeated 5 times. Then, the mixture was centrifuged at 700 g for 10 min at 4 ℃ and the resulting cell suspensions was further centrifuged at 14000 g for 30 min at 4 ℃. The precipitate was resuspended in deionized water and stored at -80 °C. The protein concentration of CM was measured using bicinchoninic acid (BCA) protein assay kit.

***Preparation of mRNA@CaCO_3_ NPs***

The CaCO_3_ NPs loaded with mRNA (mRNA@CaCO_3_ NPs) were prepared in a water-in-oil reverse microemulsion^2, 3^. Briefly, 50 μg of mRNA was added to 600 μL of 2.5 M CaCl_2_, which was subsequently dispersed in 20 mL of cyclohexane/triton-X100/hexanol (75:15:10, V:V:V) oil phase to form the “calcium microemulsion”. 600 μL of 12.5 mM Na_2_CO_3_ was dispersed in a second oil phase to form the ‘‘carbonate microemulsion”. The two microemulsions were mixed and stirred at room temperature for 45 min, Then, 40 mL of ethanol was added to break the microemulsion. The mRNA@CaCO_3_ NPs were collected with centrifugation at 12,000g for 30 min and then washed twice with 40 mL of ethanol to remove cyclohexane and surfactant. mRNA encapsulation efficiency was calculated via RiboGreen assay.

***Preparation of mRNA@CM-CaCO_3_ NPs***

mRNA@CM-CaCO_3_ NPs were prepared through coating mRNA@CaCO_3_ NPs with GL261 CM using a co-extrusion method^4^. In brief, the CM solution (containing 0.5 mg protein) was mixed with mRNA@CaCO_3_ NPs solution (containing 0.5 mg mRNA). Then the mixtures were co-extruded with a 200 nm polycarbonate membrane for 11 passes to prepare mRNA@CM-CaCO_3_ NPs.

***Preparation of*** ***mRNA@cRGD-CM-CaCO_3_ NPs***

First, endo BCN-cRGD was synthesized by mixture of endo-BCN (30 mg), cRGD-NH_2_ (100 mg), 0.15 mL triethylamine (TEA) in 1 mL dimethylformamide (DMF). After reaction at room temperature for 2 h, the reaction product endo BCN-cRGD was purified by high-performance liquid chromatography (HPLC, Agilent Inifinity1220, Germany) and confirmed by mass spectrometry (BRUKER micrOTOF II, Germany). The synthesized endo BCN-cRGD was then mixed with mRNA@cRGD-CM-CaCO_3_ NPs attached by azide groups for 1 h reaction at room temperature to form the final mRNA@cRGD-CM-CaCO_3_ NPs.

***Characterization of NPs***

The hydrodynamic sizes and zeta potentials of NPs were measured by dynamic light scattering (Zetasizer Nano ZS, Malvern Instruments Ltd UK). The morphologies of NPs were observed by transmission electron microscope (TEM, JEOL 100CX II, Japan). The energy-dispersive X-ray spectroscopy (EDS) elemental mapping was acquired using a field emission transmission electron microscope (FTEM, Talos F200X, USA).

***In vitro pH-triggered mRNA release and CO_2_ generation***

pH-dependent release of mRNA and CO_2_ generation from mRNA@cRGD-CM-CaCO_3_ NPs were studied using a dialysis method at 37 ℃. Cy3-labelled mRNA was used for quantitative analyses of mRNA concentration. pH 7.4 and 5.5 phosphate buffered saline (PBS) solutions were used as the media to simulate normal blood/tissue and the tumoral lysosome acidic condition^5^. The mRNA@cRGD-CM-CaCO_3_ NPs (containing 1 mg CaCO_3_) were placed into pretreated dialysis bags (MW cutoff 14 kDa). The dialysis bags were placed into brown bottles containing 100 mL of PBS solutions of different pH values. These bottles were shaken at 37 ℃ while shielded from light. Samples were withdrawn at various intervals and replaced with an equal volume of fresh buffer. The amount of released Cy3-mRNA was analyzed by SpectraMax M5 microplate reader (Molecular Devices, LLC., Sunnyvale, CA). The of CO_2_ concentration in PBS solutions was determined by a dissolved carbon dioxide meter (Fenglin Technology, PXS-CO2, China).

***In Vitro homotypic targeting study***

HepG2 cells, 4T1 cells, CT26 cells, and GL261 cells were separately incubated with Cy3-mRNA@CM-CaCO_3_ NPs or Cy3-mRNA@cRGD-CM-CaCO_3_ NPs (500 ng mRNA/well) for 8 h in 12-well plates. After washing and suspending in PBS, the average fluorescence of cells in each well was measured by a flow cytometer (FACS, LSRII, BD).

***mRNA*** ***transfection efficiency and cytotoxic activities***

First, mRNA encoding firefly luciferase (FLuc mRNA) was used to evaluate the mRNA transfection efficiency of NPs. GL261 cells were seeded in white 96-well plates at the density of 2 × 10^4^ cells per well, cultured overnight, and then treated with FLuc mRNA@cRGD-CM-CaCO_3_ NPs or FLuc mRNA@cRGD-CM-CaCO_3_ NPs plus US irradiation at the concentration of 50 ng mRNA/well. After 18 h, 100 μL luciferase substrate was added to each well. After 5 min, the luminescence intensity was measured by the SpectraMax M5 microplate reader.

Cell viability was determined by MTT assay. GL261 Cells were seeded in 96-well plates at 2 × 10^4^ cells per well for overnight. After 24 h of treatment with PBS, empty cRGD-CM-CaCO_3_ NPs, empty cRGD-CM-CaCO_3_ NPs plus US, IL-12 mRNA@cRGD-CM-CaCO_3_ NPs, or IL-12 mRNA@cRGD-CM-CaCO_3_ NPs plus US (50 ng mRNA/well), MTT solution was added. After an additional 4 h incubation, the supernatants were removed carefully and followed by the addition of 150 μL per well of DMSO. Absorbance was measured at 570 nm using the SpectraMax M5 microplate reader.

***Assessment of extracellular DAMPs Levels***

For measuring extracellular high mobility group protein 1 (HMGB1) and adenosine triphosphate (ATP) level, GL261 cells were seeded in a 6-well plate (5.0 × 10^5^ cells/well) and incubated with PBS, empty cRGD-CM-CaCO_3_ NPs, empty cRGD-CM-CaCO_3_ NPs plus US, IL-12 mRNA@cRGD-CM-CaCO_3_ NPs, or IL-12 mRNA@cRGD-CM-CaCO_3_ NPs plus US at the IL-12 mRNA concentration of 1 μg/well for 10 h. The supernatant was collected and measured via corresponding ELISA kit.

***Bone marrow-derived dendritic cell (BMDC) maturation***

BMDCs were isolated from C57BL/6 mice as previously described^6^. The BMDCs were co-incubated with treated GL261 cells for 24 h. Then, BMDCs were harvested and stained with anti-CD11c antibody as DC marker and anti-CD86 antibody as maturation marker. Finally, the cells were analyzed by FACS.

***Animals model***

C57BL/6 mice (5-6 weeks old, female) were purchased from Beijing Vital River Laboratory Animal Technology Company (Beijing, China). The experimental protocol was approved by Committee on Ethical Animal Experiment at Huazhong University of Science and Technology.

To construct the GL261 intracranial orthotopic glioblastoma mice model, C57BL/6 mice were anesthetized with isoflurane and set in a stereotactic instrument. Then 1.0 × 10^5^ GL261 cells in a 5 µL volume were injected into the right striatum (1 mm anterior, 2 mm right lateral from bregma, and 3.5 mm deep). The skin was closed using surgical glue.

***In vivo biodistribution study***

The study was manipulated on GL261 intracranial orthotopic glioblastoma mice model. FLuc mRNA loaded CaCO_3_ NPs were prepared. Luc mRNA@CaCO_3_ NPs, Luc mRNA@CM-CaCO_3_ NPs, Luc mRNA@cRGD-CM-CaCO_3_ NPs were intravenously injected at a dose of 0.25 mg/kg mRNA. 6 h later, mice were intraperitoneally injected with the D-luciferin substrate (30 mg/mL). 8 min post injection, bioluminescence signals were immediately measured using a IVIS Spectrum system (PerkinElmer).

***In vivo toxicity evaluation***

To evaluate the acute toxicity of the nanoparticles, healthy C57BL/6 mice received injections of PBS, IL-12 mRNA@CaCO_3_ NPs, IL-12 mRNA@CaCO_3_ NPs + US, IL-12 mRNA@cRGD-CM-CaCO_3_ NPs, IL-12 mRNA@cRGD-CM-CaCO_3_ NPs + US. After that, the blood samples of the mice were harvested. The quantities of alanine transaminase, aspartate transaminase, blood urea nitrogen and creatinine were determined using the corresponding assay kits. Histological analyses on the normal organs, including the heart, liver, spleen, lung and kidney, using haematoxylin and eosin (H&E) staining were performed, finally imaged by an optical microscope.

***In vivo antitumor efficacy***

GL261 cells were transformed with luciferase gene (GL261-Luc). And the *in vivo* antitumor study was manipulated on GL261-luc intracranial orthotopic glioblastoma mice model. The real-time bioluminescence imaging was used to evaluate the therapeutic efficiency of different types of formulation after tumor implantation. The GL261-luc mice were randomly divided into five groups (n = 5 per group), which were intravenously injected with 5 injections of PBS, IL-12 mRNA@CaCO_3_ NPs, IL-12 mRNA@CaCO_3_ NPs + US, IL-12 mRNA@cRGD-CM-CaCO_3_ NPs, IL-12 mRNA@cRGD-CM-CaCO_3_ NPs + US on day 5, 8, 11, 14, and 17 after implantation, containing IL-12 mRNA (0.5 mg kg^−1^) per dose. The bioluminescence signal was used to evaluate the therapeutic efficiency at 5th, 10th,15th and 20th day after tumor implantation through the IVIS Spectrum system. Throughout the study, mice were weighted regularly.

***ELISA and immunofluorescence***

The isolated brains were collected and ground in tissue protein extraction reagent in the presence of 1% proteinase and phosphatase inhibitors. The lysates were incubated at 4 °C for 30 min with slow rotation then centrifuged to remove debris. The supernatants were used for IFN-γ, IL-12 ELISA analysis. For immunofluorescence, the isolated brains were first fixed within 4% paraformaldehyde overnight, followed with the dehydration by 15% and 30% sucrose solution for another 24 h in sequence and stained with CD8 antibody with dilution ratio 1:100. The sections were further stained with corresponding secondary antibody and DAPI. Images were captured using a fluorescence microscope (Olympus SZX12, Japan).

***Statistical analysis***

All data were taken from three independent experiments and then expressed as means ± standard error of mean (SEM). Statistical significance was analyzed using an unpaired, two-tailed Student’s t-test, one-way or two-way ANOVA analysis by GraphPad Prism 5.0 (San Diego, CA, USA). Statistical significance thresholds were set at *p < 0.05, **p < 0.01, ***p < 0.001.

**References**

1. Duan, Y.; Wu, M.; Hu, D.; Pan, Y.; Hu, F.; Liu, X.; Thakor, N.; Ng, W. H.; Liu, X.; Sheng, Z.; Zheng, H.; Liu, B., Biomimetic Nanocomposites Cloaked with Bioorthogonally Labeled Glioblastoma Cell Membrane for Targeted Multimodal Imaging of Brain Tumors. *Advanced Functional Materials* **2020,** *30* (38), 2004346.

2. Kim, S. K.; Foote, M. B.; Huang, L., Targeted delivery of EV peptide to tumor cell cytoplasm using lipid coated calcium carbonate nanoparticles. *Cancer Letters* **2013,** *334* (2), 311-318.

3. Li, J.; Yang, Y.; Huang, L., Calcium phosphate nanoparticles with an asymmetric lipid bilayer coating for siRNA delivery to the tumor. *Journal of Controlled Release* **2012,** *158* (1), 108-114.

4. Wu, M.; Liu, X.; Bai, H.; Lai, L.; Chen, Q.; Huang, G.; Liu, B.; Tang, G., Surface-Layer Protein-Enhanced Immunotherapy Based on Cell Membrane-Coated Nanoparticles for the Effective Inhibition of Tumor Growth and Metastasis. *ACS Applied Materials & Interfaces* **2019,** *11* (10), 9850-9859.

5. Lim, E.-K.; Huh, Y.-M.; Yang, J.; Lee, K.; Suh, J.-S.; Haam, S., pH-Triggered Drug-Releasing Magnetic Nanoparticles for Cancer Therapy Guided by Molecular Imaging by MRI. *Advanced Materials* **2011,** *23* (21), 2436-2442.

6. Um, W.; Ko, H.; You, D. G.; Lim, S.; Kwak, G.; Shim, M. K.; Yang, S.; Lee, J.; Song, Y.; Kim, K.; Park, J. H., Necroptosis-Inducible Polymeric Nanobubbles for Enhanced Cancer Sonoimmunotherapy. *Advanced Materials* **2020,** *32* (16), 1907953.


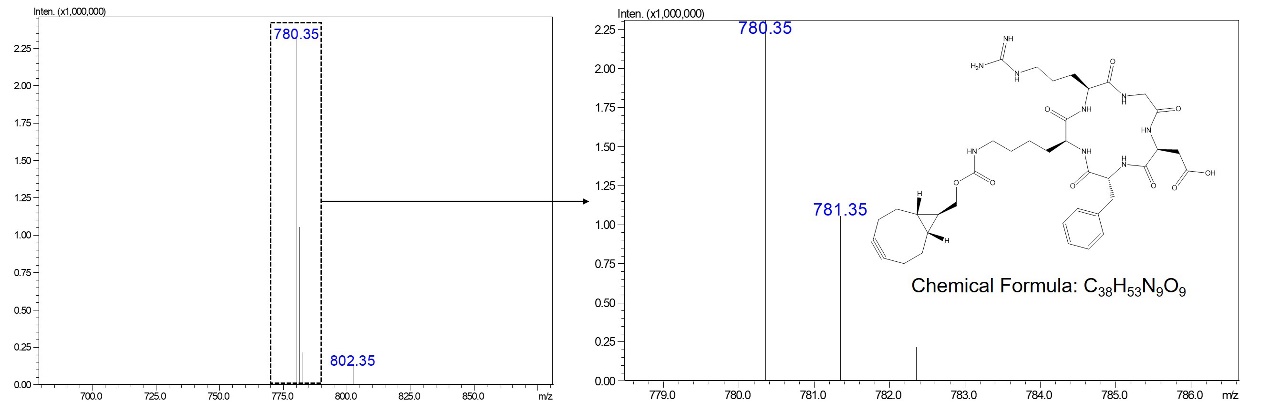


**Figure S1.** Mass measurement of synthesized endo-BCN-Crgd.

**
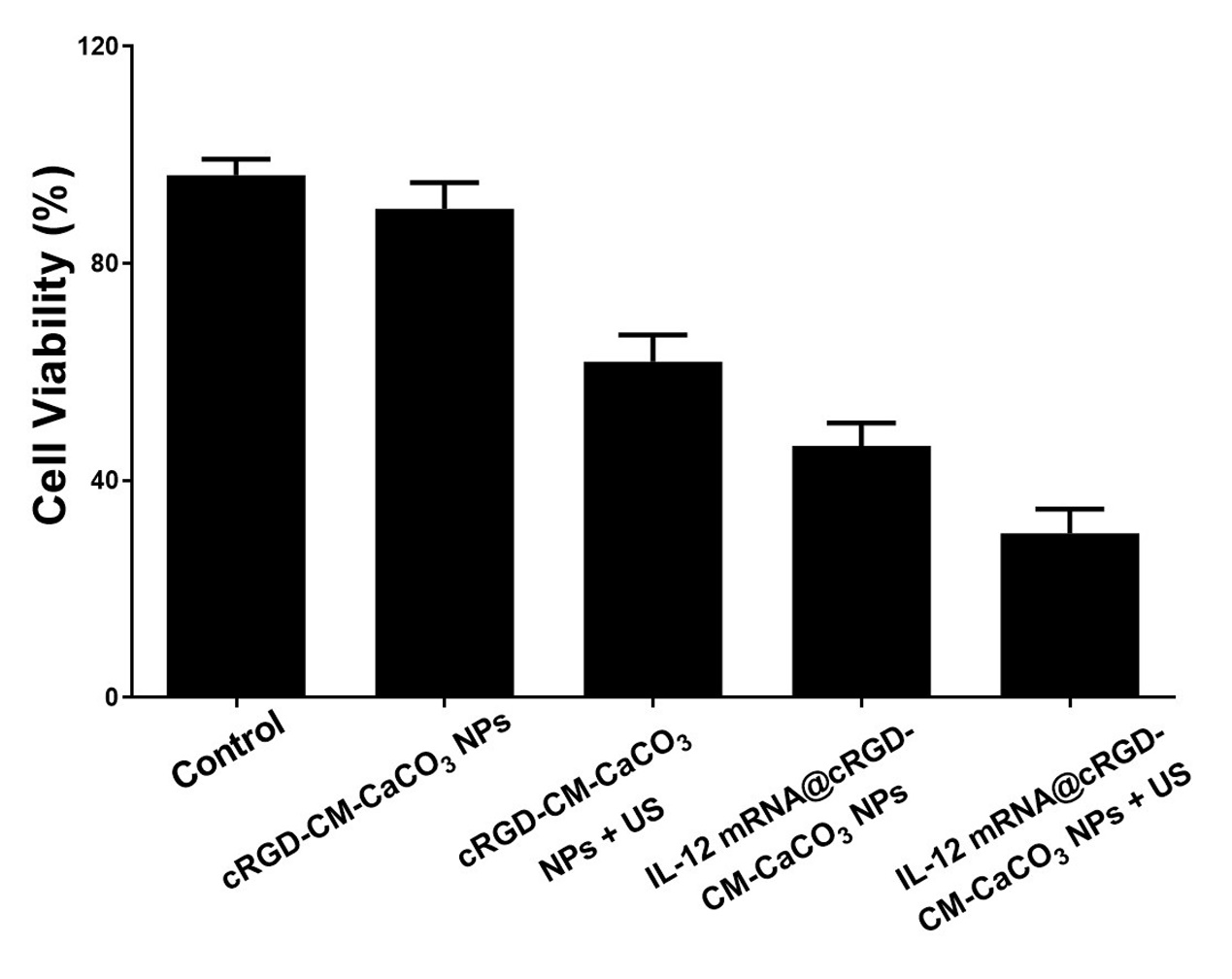
**

**Figure S2.** Viabilities of GL261 cells after different treatment for 24 h.


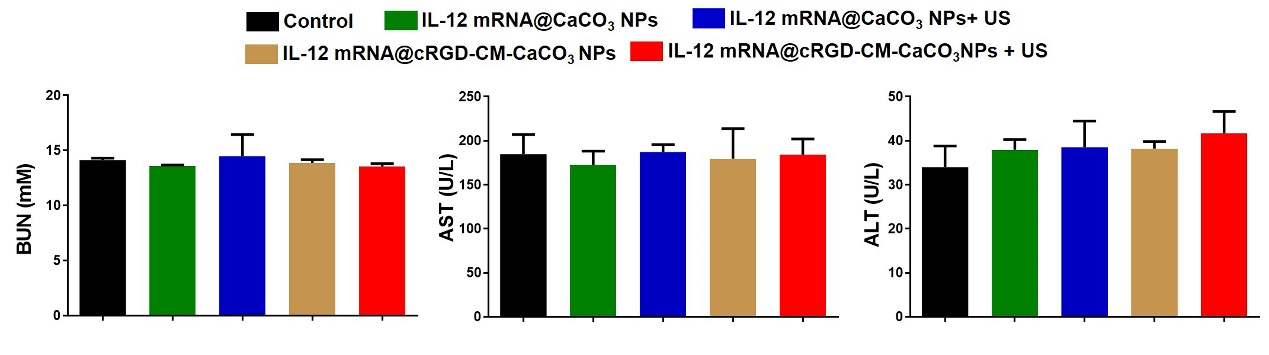


**Figure S3.** Effect of different treatments on serum BUN, AST and ALT levels.


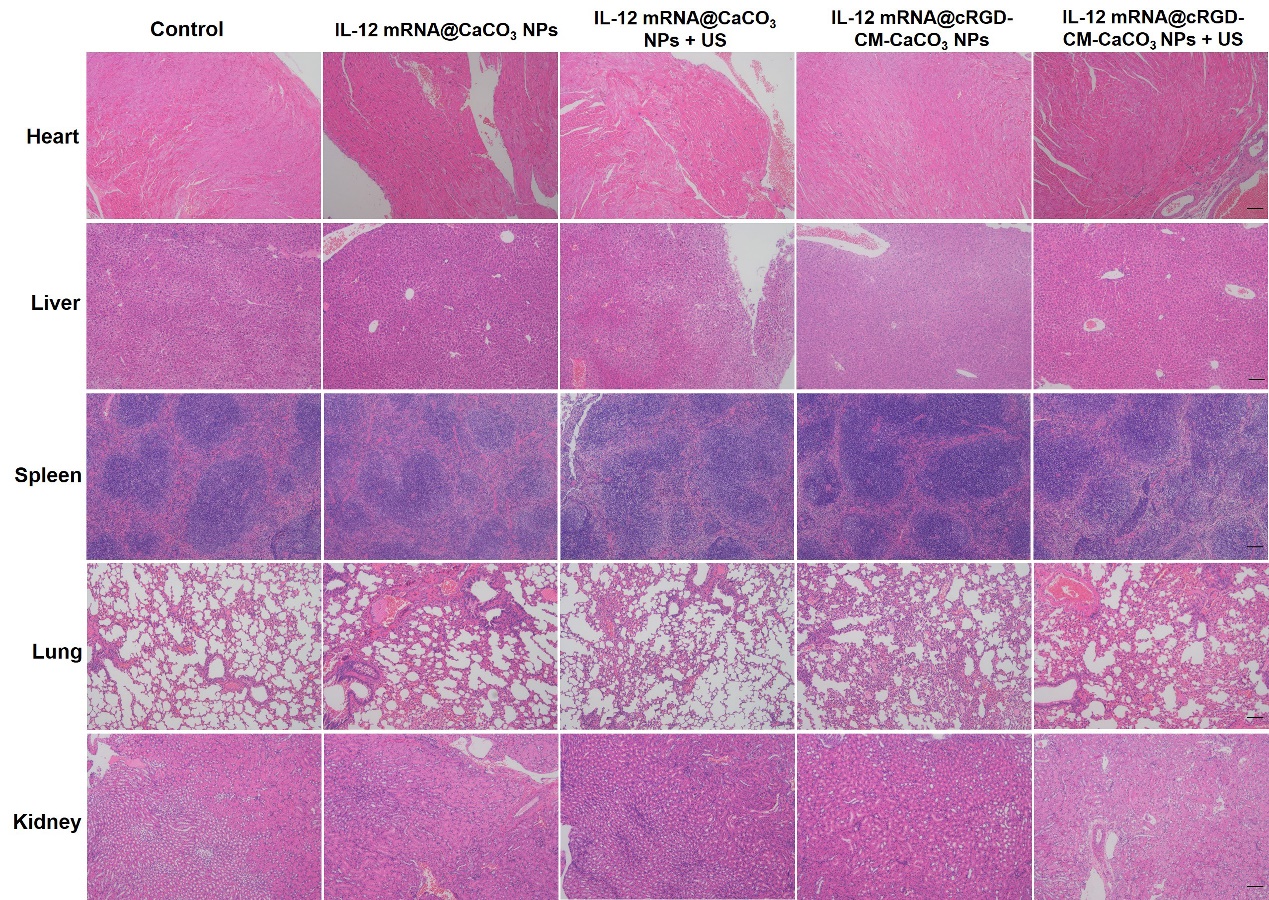


**Figure S4.** H&E staining of major organs after treatments. Scale bar, 100 µm.


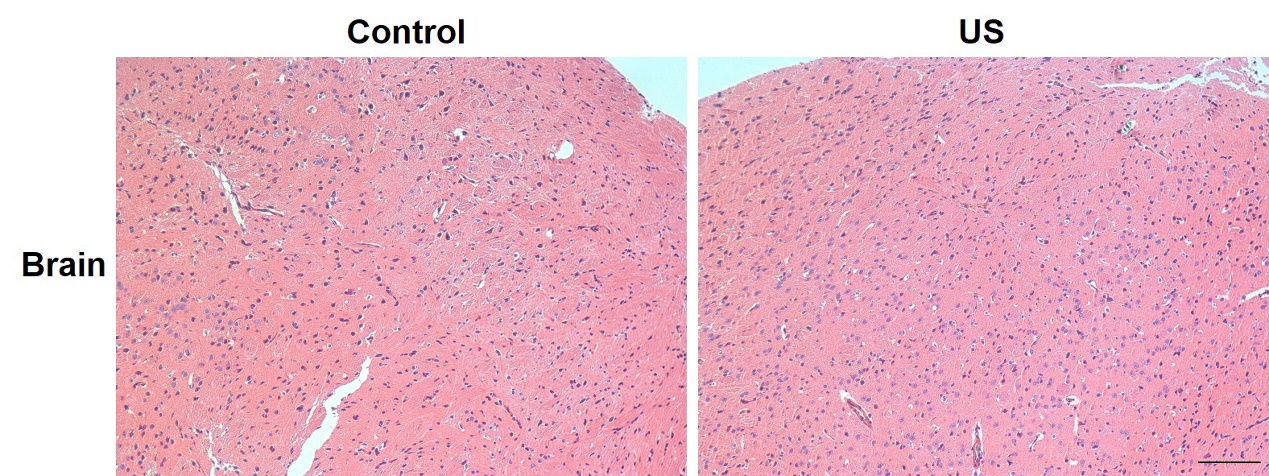


**Figure S5.** H&E staining of brain after US irradiation. Scale bar, 100 µm.


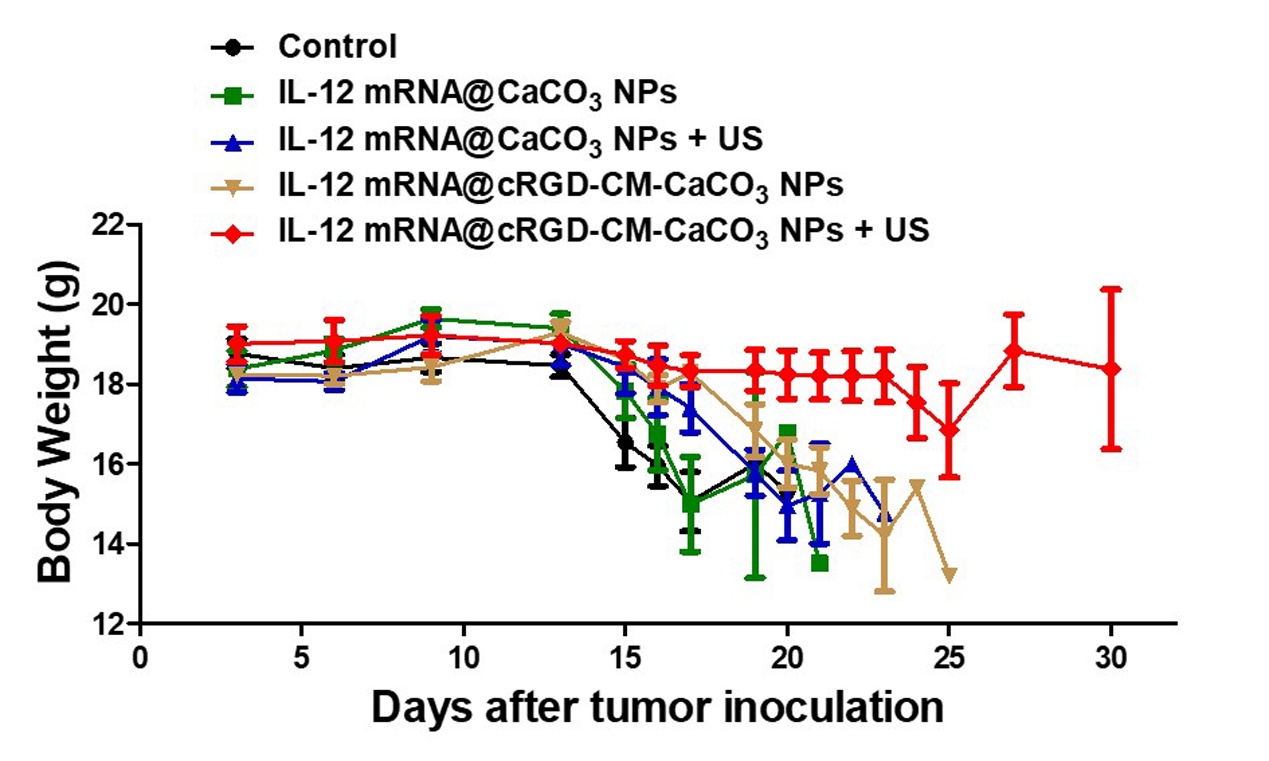


**Figure S6.** Body weight change of different groups.
